# Developing foundations for biomedical knowledgebases from literature using large language models – A systematic assessment

**DOI:** 10.1016/j.csbj.2025.07.042

**Published:** 2025-07-24

**Authors:** Chen Miao, Zhenghao Zhang, Jiamin Chen, Daniel Rebibo, Haoran Wu, Sin-Hang Fung, Alfred Sze-Lok Cheng, Stephen Kwok-Wing Tsui, Sanju Sinha, Qin Cao, Kevin Y. Yip

**Affiliations:** a School of Biomedical Sciences, The Chinese University of Hong Kong, Shatin, New Territories, Hong Kong; b Department of Computer Science and Engineering, The Chinese University of Hong Kong, Shatin, New Territories, Hong Kong; c Center for Data Sciences, Sanford Burnham Prebys Medical Discovery Institute, La Jolla, CA, USA; d Cancer Genome and Epigenetics Program, NCI-Designated Cancer Center, Sanford Burnham Prebys Medical Discovery Institute, La Jolla, CA, USA; e Cancer Metabolism and Microenvironment Program, NCI-Designated Cancer Center, Sanford Burnham Prebys Medical Discovery Institute, La Jolla, CA, USA; f Center for Therapeutics Discovery, Sanford Burnham Prebys Medical Discovery Institute, La Jolla, CA, USA; g Shenzhen Research Institute, The Chinese University of Hong Kong, Shenzhen, China; h Center for Neurologic Diseases, Sanford Burnham Prebys Medical Discovery Institute, La Jolla, CA, USA

**Keywords:** Large language models, Biomedical knowledgebases, Prompt engineering

## Abstract

While large language models (LLMs) have shown promising capabilities in biomedical applications, measuring their reliability in knowledge extraction remains a challenge. We developed a benchmark to compare LLMs in 11 literature knowledge extraction tasks that are foundational to automatic knowledgebase development, with or without task-specific examples supplied. We found large variation across the LLMs’ performance, depending on the level of technical specialization, difficulty of tasks, scattering of original information, and format and terminology standardization requirements. We also found that asking the LLMs to provide the source text behind their answers is useful for overcoming some key challenges, but that specifying this requirement in the prompt is difficult.

## 1 Introduction

Knowledgebases are structured repositories that systematically organize and store knowledge about entities and their relationships, designed to provide efficient access to accurate, standardized, and up-to-date information [1]. A key information source of biomedical knowledgebases is the scientific literature, which describes knowledge primarily in free text. While extracting, organizing, and annotating knowledge from the literature has long been assisted by computational methods [2], [3], [4], [5], the recent emergence of large language models (LLMs) has offered new opportunities in automated knowledgebase development [6], [7], [8], [9], [10], [11] for two reasons. First, LLMs can generate new text, which is useful for summarizing extracted knowledge in easily understandable forms. Second, LLMs can handle tasks that they are not specifically designed for, which means LLMs trained on general text corpuses can potentially handle highly specialized biomedical topics.

However, some major concerns about the use of LLMs in biomedicine remain [12], including 1) accuracy, that information contained in an LLM can come from unvalidated sources, 2) hallucination, that the text generated can be fabricated and not actually factual, and 3) transparency and interpretability, that LLMs are mostly “black boxes” that produce answers in unexplained ways.

Here we propose methodologies for using LLMs to develop biomedical knowledgebases and systematically evaluate their performance in terms of information extraction accuracy, completeness, and consistency. To assess LLMs’ abilities in handling highly specialized knowledge, we focus on the specific topic of biomarkers that indicate efficacy of cancer immune checkpoint inhibition (ICI) therapies. We choose this topic because 1) ICI treatment outcome varies substantially across cancer types and patients [13], [14], leading to publications of many efficacy biomarkers [15], [16], [17] that require a knowledgebase to organize, and 2) each biomarker is described by both simple (e.g., direction of efficacy association) and complex (e.g., experiments required) attributes, which naturally define tasks of varying difficulties for the LLMs. Generalizing from this case study, we provide insights applicable to other biomedical knowledgebases.

## 2 Results and discussion

### 2.1 Benchmark and study designs

**Fig. 1:**
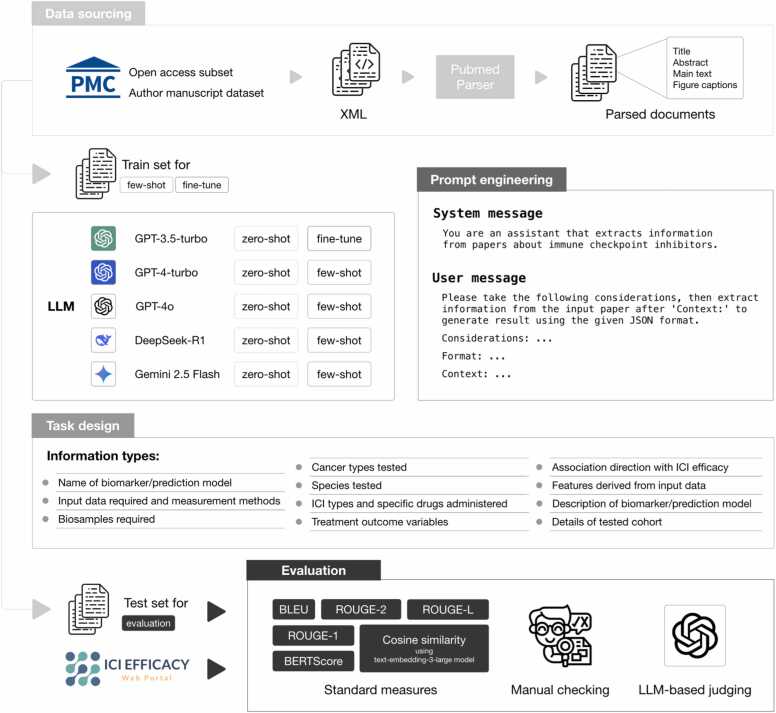
Overview of our workflow. Publications obtained from PubMed Central were split into training and test sets. Five different LLMs were compared based on their performance on 11 tasks. Each LLM was used in both the zero-shot and few-shot/fine-tuned zero-shot settings. For the latter, the task-specific examples were derived from the training set. Answers produced by the LLMs about publications in the test set were evaluated using three different strategies.

We previously developed a manually curated knowledgebase about ICI efficacy biomarkers and prediction models by reading relevant publications, extracting knowledge from them, and summarizing the knowledge using standard formats and terminologies (https://iciefficacy.org/) [18]. Among the types of knowledge collected, we selected 11 in this study to test whether LLMs could extract them from the publications correctly (Fig. 1). These 11 types of knowledge vary substantially in data type and content complexity (Fig. 1, **Methods**).

We designed prompts to specify the tasks, expected formats, and text of the publications that report the biomarkers (Fig. 1, **Methods**). For three “definition question” tasks that request a definition, we also asked the LLMs to provide the specific parts of the source text behind their answers. We compared five LLMs, namely GPT-3.5 turbo, GPT-4 turbo, GPT-4o, DeepSeek-R1, and Gemini 2.5 Flash. For each LLM, we tested both the zero-shot and few-shot/fine-tuned zero-shot settings to evaluate the benefits of providing task-specific examples (**Methods**). Therefore, in total we had ten contestants.

We used three different strategies to systematically evaluate the contestants’ performance (**Methods**). First, we compared their answers with our manual curation results and quantified their similarity using six standard performance measures (BLEU [19], ROUGE-1 [20], ROUGE-2 [20], ROUGE-L [20], BERTScore [21], and Cosine similarity). Second, we asked two human inspectors to independently score the contestants’ answers based on a standardized scoring scheme and then averaged their scores. Third, we supplied both the contestants’ answers and the manual curation results as inputs to an LLM and asked it to score those answers.

### 2.2 Dependency of performance on LLMs and availability of task-specific examples

**Fig. 2:**
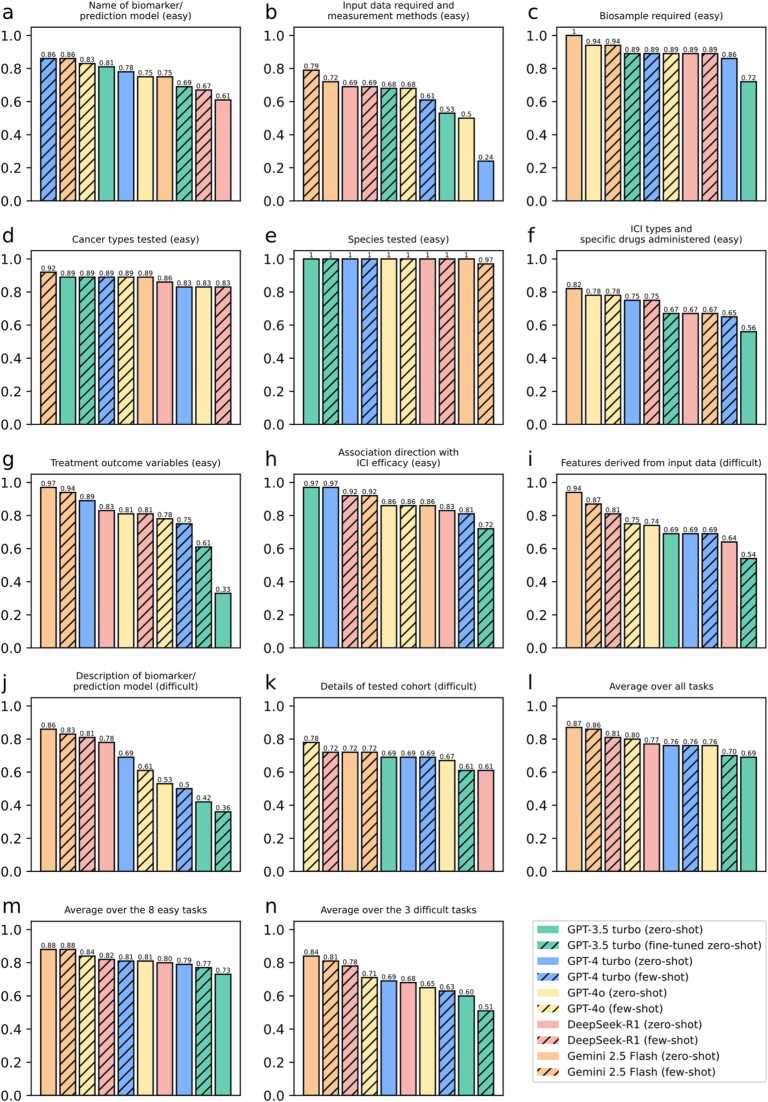
**Performance of the LLM based on our manual checking scores.** The different panels show the scores of individual tasks (**a-k**), and the average scores over all tasks (**l**), 8 easy tasks (**m**), and 3 difficult tasks (**n**). In case of a tie in performance scores, models are shown according to a predefined order: GPT-3.5 turbo, followed by GPT-4 turbo, GPT-4o, DeepSeek-R1, and Gemini 2.5 Flash. For ties between settings for the same model, the zero-shot setting is shown first.

The full set of results is provided in Supplementary Materials. Here we give an overview of the contestants’ performance based on the human inspectors’ average scores (0–1, where higher is better), which we found to capture contextual and semantic information better than the other two evaluation strategies (this observation will be elaborated below). Fig. 2 shows the scores of the 10 contestants in each of the 11 tasks (Fig. 2**a-k**), and their average scores over i) all 11 tasks (Fig. 2**l**), ii) 8 easy tasks (Fig. 2**m**), and iii) 3 difficult tasks (Fig. 2**n**).

Judged by the average scores, newer LLMs (GPT-4o, GPT-4 turbo, DeepSeek-R1, and Gemini 2.5 Flash) tended to outperform an older LLM (GPT-3.5 turbo) in both easy and difficult tasks. However, the average performance scores of the 10 contestants showed substantial variability between the 11 evaluated tasks, and no single contestant consistently outperformed the others.

Comparing the tasks, the one that the contestants performed the best was “Species tested”, with most contestants getting full scores (Fig. 2**e**). Overall, the contestants achieved higher scores in the easy tasks than in the difficult tasks (Fig. 2**m,n**).

Comparing zero-shot and few-shot/fine-tuning, in the 5 (LLMs) × 11 (tasks) = 55 comparisons, the latter received a higher score only 24 times and tied with the former 12 times. In the remaining 19 times, few-shot/fine-tuning led to a lower score than zero-shot. Therefore, in this study, supplying task-specific examples did not result in coherent performance improvements. For example, these examples were generally not helpful for tasks that involve a large variation of writing style among publications (e.g., “Description of biomarker/prediction model”, Fig. 2**j**) or that require interpreting the text rather than straightforward text extraction (e.g., “Association direction with ICI efficacy”, Fig. 2**h**) because the LLMs failed to generalize from a few task-specific examples.

Comparing the average scores over all tasks, the 8 easy tasks, and the 3 difficult tasks, Gemini 2.5 Flash was the model with the best performance.

### 2.3 General insights obtained by analyzing individual tasks

Next, we analyze the individual tasks in detail and make observations that provide general insights into the use of LLMs in developing biomedical knowledgebases.

First, all contestants performed better in tasks that involve basic text-parsing rather than those that involve ICI-specific knowledge. For example, all contestants achieved high scores in the “Biosamples required”, “Cancer types tested”, and “Species tested” tasks (Fig. 2**c,d,e**) but not in “ICI types and specific drugs administered” and “Treatment outcome variables” (Fig. 2**f,g**).

Second, LLMs may struggle to generate accurate answers when some relevant information is not directly contained in the publication text supplied. For example, in the “ICI types and specific drugs administered” task, LLMs frequently failed to identify the correct drugs when their names were not directly contained in the publication text supplied. This finding suggests that even when relevant information is likely part of their extensive pre-training data, such as the widely prevalent knowledge of common ICI drugs, the models may struggle to apply this latent knowledge to the specific context of the supplied text.

Third, most contestants did not demonstrate a strong ability in identifying and combining information scattered across different parts of a publication, with the notable exception of Gemini 2.5 Flash, which showed some improvement in this area. For example, in the “Input data required and measurement methods” task, the other LLMs often could not generate a complete answer that involves information from different Results and Methods subsections of a publication, whereas Gemini was partially successful.

Fourth, some tasks are complex and thus it is challenging to fully specify them using a standardized prompt. For example, for the “Description of biomarker/prediction model” task, in order to produce a description that uses a common format and standard terminologies, the prompt needs to specify many requirements. Otherwise, post-processing of the answer is necessary, possibly using follow-up prompts. While the GPT models struggled to generate satisfactory results under these constraints, the newer models with explicit reasoning capabilities, namely DeepSeek-R1 and Gemini 2.5 Flash, proved to be significantly more capable of producing a compliant output directly.

Fifth, few-shot did not consistently outperform zero-shot. In our study, this appeared to be the case especially for tasks involving large variations in writing style or requiring deep interpretation rather than straightforward text extraction. Our finding aligns with existing research on the “brittleness” of few-shot learning, which is an active area of investigation. The literature primarily attributes this phenomenon to three potential reasons, namely models overfitting to superficial patterns in misleading examples [22], high sensitivity to prompt construction such as example order and format [23], [24], and the insufficiency of a few demonstrations to fully specify a complex task [25].

Sixth, asking an LLM for the source text behind its answers is a useful idea but difficult to implement. The source text can help address all three major concerns described above (accuracy, hallucination, and transparency/interpretability) by making it easier to check where the information comes from and whether the LLM summarizes it accurately. However, the LLMs frequently ignored our request, and rarely could they return source text that comes from multiple parts of a publication.

Seventh, the six standard performance measures showed low consistency with the manually checked scores (Supplementary Figure 1). This phenomenon is also observed in different studies across video captioning [26], medical evidence summarization [27], and abstractive summarization systems [28]. These measures have been commonly used in natural language processing (NLP) [29], [30], [31] partly because they can be computed automatically, and are thus easily applicable to comparing many methods in many tasks. However, we found that they focused too much on token similarity but failed to capture contextual information well (Supplementary Materials). For example, BLEU and ROUGE tended to give a low score to correct but lengthy answers, but a high score to concise though incomplete answers (Supplementary Table 1). Likewise, asking an LLM to judge the similarity between an answer and the reference (Evaluation Strategy 3) also led to results inconsistent with manual checking (Supplementary Figure 1).

Eighth, Gemini 2.5 Flash achieved the highest average performance across the 11 evaluated tasks. This superior overall result suggests that its reasoning capabilities may be a key factor, indicating that further development of this ability is a critical path to elevating future model performance.

## 3 Conclusions

We conclude that LLMs can be very helpful in assisting in the development of biomedical knowledgebases but full automation is not yet feasible. Major barriers include 1) extracting and summarizing information from different parts of a publication, 2) handling specialized topics with no/few examples, and 3) standardizing terminologies and formats. If LLMs’ answers are to be manually verified, having them return the source text behind their answers would be very useful. For this strategy, major difficulties to overcome include specifying the requirement in the prompt and quantitative measures that can judge how well the LLMs’ answers summarize the source text. Overall, the benchmark we established here can help systematically compare LLMs in future studies. Our benchmark complements other valuable benchmarks [32], [33], including those assessing symbolic or combinatorial reasoning, forming a varied and essential toolkit for advancing LLMs from general-purpose tools into reliable, domain-specific solutions. Beyond our assessment of general-purpose LLMs, the field is rapidly advancing through diverse, specialized approaches; pioneering efforts in agent-based systems (e.g., Crow from FutureHouse), multi-LLM frameworks [34], and domain-specific models [35], [36] are collectively paving the way toward the reliable automation of biomedical knowledgebase curation (Supplementary Text).

## 4 Methods

### 4.1 Details of the 11 tasks

We defined 11 tasks to evaluate LLMs’ abilities in extracting different types of information from a publication and summarizing them into a form suitable for a knowledgebase. The 11 tasks are:

1. Name of biomarker/prediction model: Determining the name of the biomarker/ prediction model proposed in the publication
2. Input data required and measurement methods: Determining the types of data required by the biomarker/prediction model (e.g., expression levels of certain genes) and the experimental methods used for the measurements (e.g., RNA sequencing)
3. Biosamples required: Determining the biosamples required by the biomarker/prediction model (e.g., cancer biopsies)
4. Cancer types tested: Determining the cancer types for which effectiveness of the biomarker/prediction model has been tested
5. Species tested: Determining the species in which effectiveness of the biomarker/prediction model has been tested
6. ICI types and specific drugs administered: Determining the ICI types (e.g., anti-PD-1) and specific drugs (e.g., pembrolizumab) that have been administered in the cohorts tested
7. Treatment outcome variables: Determining the variables used to quantify treatment outcome (e.g., progression-free survival)
8. Association direction with ICI efficacy: Determining whether the biomarker/prediction model is positively or negatively associated with favorable ICI treatment outcome
9. Features derived from input data: Describing the features derived from the input data that are subsequently used to define the biomarker/prediction model
10. Description of biomarker/prediction model: Describing the exact definition of methodologies used to construct the biomarker/prediction model and its connection to ICI efficacy
11. Details of tested cohort: Describing details of the cohort studied (e.g., number of samples and other treatments previously received)

We defined tasks 1–8 as easy and 9–11 as difficult. This characterization was based on a) length of answer, b) whether there is a limited set of possible answers, and c) whether answers need to be extracted and consolidated from multiple parts of the manuscript.

### 4.2 Publications involved in this study

From our ICI efficacy knowledgebase [18], we selected 29 publications that propose biomarkers/prediction models of ICI treatment outcome (Supplementary Table 2). These publications were selected because they had full text freely available, and they described a variety of biomarkers/prediction models.

For each of these publications, we have previously performed all 11 tasks (as well as other tasks) by manually reading the publications, extracting the information, and summarizing them using standard formats and terminologies [18]. These manual curation results were used as reference for evaluating the performance of the LLMs’ answers.

We obtained full text of the 29 publications from either the PubMed Central (PMC) Open Access (OA) Subset, using its application programming interface (API) at https://www.ncbi.nlm.nih.gov/pmc/utils/oa/oa.fcgi?id= [PMCID], or the PMC Author Manuscript Dataset, using its file transfer protocol (FTP) site at https://ftp.ncbi.nlm.nih.gov/pub/pmc/manuscript/xml/. In both cases, the full text was obtained in extensible markup language (XML) format.

We parsed the XML files using the Pubmed Parser package (version 0.4.0) [37]. For each publication, we extracted its title, abstract, main text, and figure captions, and concatenated them into a single text string.

Both the fine-tuned and few-shot settings (detailed below) require a training set of publications with “ground truth” answers to the tasks. We randomly split the 29 publications into a training set of 20 publications and left the remaining 9 publications as a test set for unbiased evaluation of the LLMs’ performance.

### 4.3 LLMs and settings

We evaluated the performance of five LLMs (Supplementary Table 3), namely GPT-3.5 turbo (gpt-3.5-turbo-0125), GPT-4 turbo (gpt-4-turbo-2024–04–09), GPT-4o (gpt-4o-2024–05–13), DeepSeek-R1 (DeepSeek-R1–0528) and Gemini 2.5 Flash (gemini-2.5-flash-preview-05–20).

For each of these LLMs, we tested two different settings:

• GPT-3.5 turbo: zero-shot and fine-tuned zero-shot
• GPT-4 turbo: zero-shot and few-shot
• GPT-4o: zero-shot and few-shot
• DeepSeek-R1: zero-shot and few-shot
• Gemini 2.5 Flash: zero-shot and few-shot

We did not test the few-shot setting of GPT-3.5 turbo because it limited the input to no more than ∼16,000 tokens, which was not enough to provide the task-specific examples. We also did not test the fine-tuned setting of GPT-4 turbo and GPT-4o because fine-tuning these models was not available at that time. To ensure a direct comparison with GPT-4 turbo and GPT-4o, we tested zero-shot and few-shot for DeepSeek-R1 and Gemini 2.5 Flash. Furthermore, we leveraged the reasoning capabilities of the two reasoning models: DeepSeek-R1 and Gemini 2.5 Flash. These capabilities, which function as an implicit form of Chain-of-Thought (CoT) [38], [39], allowed us to investigate whether our single-prompt design was effective at engaging the models’ powerful internal reasoning processes and thereby addressing the need for multi-step reasoning.

For the fine-tuned and few-shot settings, we extracted the manually curated answer of each task for the publications in our training set from our ICI efficacy web portal and converted it into a JavaScript Object Notation (JSON) format as the reference answers. To fine-tune a model, we used the official fine-tuning service provided by OpenAI, supplying all 20 training publications and the corresponding reference answers for 10 epochs. For the few-shot setting, we randomly selected three publications from the training set and supplied them as well as the corresponding reference answers as input to the LLMs. Across all experiments, the temperature parameter was set to 0.1. This low value was deliberately chosen to minimize output randomness, thereby focusing our analysis on the models’ core capabilities for tasks requiring high precision and factual correctness.

### 4.4 Prompt design

A prompt generally consists of three parts: a system message, a user message, and an optional previous conversation history. In this study, we only used the system message and user message in our prompts (Supplementary Figure 2). A system message is a predefined message that establishes the context or provides instructions for the model to guide the model’s responses. A user message is the input given by the user, which the model needs to respond to.

We first designed an initial prompt based on the task requirements. To optimize this prompt, we used two additional publications [17], [40] from our curated knowledgebase as a development set, which were not involved with the set of selected 29 publications. Based on the zero-shot responses from GPT-3.5 turbo on this set, we iteratively revised the prompt for all 11 tasks. For example, the vague term “Data Required” was not specific enough to reflect the exact information we wanted to extract, and thus we added more specific descriptions in the “Considerations” section: “Data Required is the input data that is required for the calculation of biomarkers or features, e.g., Protein Level – ELISA Test.” The final prompt, refined through this process, was then used for the main study (Supplementary Figure 2). For all the tasks, we used the system message to specify the top-level instructions. Additionally, for three tasks (“Name of biomarker/prediction model”, “Features derived from input data”, and “Description of biomarker/prediction model”), we used the system message to specify the requirement that the source text underlying the answer should also be returned. Specifically, the system message included was “If an answer is provided, it must be annotated with a source text that supports or provides evidence for the answer.”

In the user message, we designed three sections: namely “Considerations”, “Format”, and “Context”. “Considerations” provided the background and specified the requirements for the model’s answer. “Format” specified the formatting requirement, and we asked the model to format the answer in JSON. “Context” contained the processed text of the publication of interest.

Among the responses of GPT models, around 5 % were not strictly valid JSON objects. Instead, they contained a descriptive text followed by a JSON substring that included information for all tested tasks, which could not be automatically parsed as JSON due to the preceding text. In contrast, all responses from the two newly tested models, namely DeepSeek-R1 and Gemini 2.5 Flash, adhered strictly to JSON formatting. For the outputs under the former condition, we manually extracted all relevant information from the responses, a process that did not influence the evaluation.

### 4.5 Details of the first evaluation strategy: standard measures

For each answer produced by an LLM, we compared it with our manual curation result and quantified their similarity using six standard measures, namely BLEU, ROUGE-1, ROUGE-2, ROUGE-L, BERTScore, and Cosine similarity.

BLEU and ROUGE are both n-gram-based metrics that assess the similarity between the n-grams (i.e., a sequence of n adjacent words) of the LLM’s response and the ground truth. Based on the similarity, they assign a score between 0 and 1, where a higher score indicates higher similarity. Since the answers were generally short, we used n = 1 when computing the BLEU score, which means individual words were considered separately. For ROUGE, we reported ROUGE-1, ROUGE-2, and ROUGE-L scores. ROUGE-1 evaluates the overlap of unigrams (single words) between the generated and reference answers; ROUGE-2 assesses the overlap of bigrams (pairs of consecutive words); and ROUGE-L measures the longest common subsequence. For the “Species tested” task, all the reference answers are single words (i.e., “Human”). In such instances, where there are no bigrams to compare, the ROUGE-2 scores are calculated as zeros.

BERTScore and Cosine similarity calculate similarity based on sentence embeddings. We used the microsoft/deberta-xlarge-mnli model [41] to produce the embedding for the former and OpenAI’s text-embedding-3-large model (https://platform.openai.com/docs/guides/embeddings/embedding-models) to produce the embedding for the latter. In theory, two identical sentences should have a BERTScore and a Cosine similarity of 1. However, we noted that the packages we used for computing Cosine similarity sometimes gave a value close to, but not identical to, 1.

ROUGE-1, ROUGE-2, ROUGE-L, and BERTScore can be used to compute precision and recall of an answer as compared to the reference. All our reported values of these measures are the F-measure, which is the harmonic mean of precision and recall.

### 4.6 Details of the second evaluation strategy: manual checking

We first asked two human inspectors to independently evaluate the performance of the LLMs’ answers using the manually curated results in our knowledgebase as a reference. The two inspectors followed the same standard scoring scheme when performing their evaluations. Then for each of the 10 (contestants) × 11 (tasks) × 9 (publications in the test set) = 990 evaluations, we computed the average score among the two inspectors.

The standard scoring scheme considered both accuracy and completeness of the answers. Roughly speaking, each answer was given either a score of 0 (Inaccurate/Incomplete), 0.5 (Partially Accurate/Complete), or 1 (Fully Accurate/Complete). An exception was made for the “Features derived from input data” task due to its complexity, which involves both i) measuring, calculating, or deriving features from raw data, and ii) transforming, combining, and selecting features for the prediction models.

Specifically, the following rules were used to determine the score of an answer in each task:

1. Name of biomarker/prediction model: 1 point for full, correct predictor name or predictor descriptions; 0.5 for partial or abbreviated predictor names or vague predictor descriptions; 0 otherwise
2. Input data required and measurement methods:

• Input data required: 1 point for completely correct data type(s); 0.5 for partially correct data types which may include fewer/more data types than the reference answer; 0 otherwise
• Measurement methods: 1 point for completely correct data type(s) corresponding technologies; 0.5 for partially correct technologies or including fewer/more than the reference answer; 0 otherwise

These two scores were then averaged to give the overall score

3. Biosamples required: 1 point for exactly correct biosample type(s) required; 0.5 for incorrect details or including fewer/more biosamples than the reference answer; 0 otherwise
4. Cancer types tested: 1 point for correctly listing all cancer types involved, including cancer type specification for pan-cancer predictors; 0.5 for partially complete answer; 0 otherwise
5. Species tested: 1 point for correct species; 0.5 for partially complete species (having additional/missing species in the answer); 0 otherwise
6. ICI types and specific drugs administered:

• ICI types: 1 point for all individual/combination immune checkpoint inhibitors (target proteins) involved; 0.5 for partially correct or partially complete answer; 0 otherwise
• Drugs: 1 point for all individual/combination ICI drugs involved, including combination with non-ICI drug(s); 0.5 for partially correct or partially complete answer; 0 otherwise; NA for information not available

These two scores were then averaged to give the overall score

7. Treatment outcome variables: 1 point for all outcome measure(s); 0.5 for partially complete answer; 0 otherwise
8. Association direction with ICI efficacy: 1 point for fully correct direction matching with predictor name/description; 0.5 if it lacks necessary explanation; 0 for incorrect
9. Features derived from input data: Each feature as 1 point (0–0.5–1, three-tier for accuracy and correctness) and each feature processing/selection category as 2 points (0–1–2, three-tier for accuracy and correctness) to emphasize importance of how the features are used in the final predictor model. The final score is the percentage of the correctness
10. Description of biomarker/prediction model: 1 point for fully correct model type and association with efficacy metrics; 0.5 for partially correct or partially complete answer; 0 otherwise
11. Details of tested cohort: 1 point for all cohort reference source(s) and corresponding patient count(s); 0.5 for partially complete answer; 0 otherwise

Some illustrative examples of how the manual checking scores were determined are given in Supplementary Text.

### 4.7 Details of the third evaluation strategy: LLM-based judging

We also asked GPT-4o to judge the performance of an answer using our manual curation result as a reference. The purpose of testing this evaluation strategy is to see whether it can capture semantic and contextual information better than the standard performance measures but at the same time offer the benefit of being a fully automatic strategy without the need for manual judging.

We performed this evaluation by modifying an example in the OpenAI cookbook on how to evaluate a summarization task: https://cookbook.openai.com/examples/evaluation/how_to_eval_abstractive_summarization. We modified the prompt to suit our needs, focusing on evaluating accuracy (Supplementary Figure 3). The resulting score ranged from 0 to 5, which was then rescaled to 0–1.

### 4.8 Comparison of the three evaluation strategies

The complete set of scores based on all three evaluation strategies is provided in Supplementary Figure 4. We calculated the Pearson’s correlation coefficient (PCC) and Spearman’s rank correlation coefficient (SCC) between our manual checking scores and the other evaluation measures. Specifically, for each task, each evaluation measure generated 10 (contestants) × 9 (publications in the test set) = 90 scores, resulting in a vector of length 90. PCC and SCC were then calculated by comparing the vector from our manual checking results with that from each of the other measures. PCC and SCC could not be computed (marked as “N.A.”) in one scenario due to one vector having all 90 values the same. The scenario was: in “Species tested”, ROUGE-2 yielded 0 s for all nine publications.

## Ethics approval and consent to participate

No ethical approval was required for this study. All utilized public datasets were generated by other organizations that obtained ethical approval.

## Funding

QC is supported by 10.13039/501100001809National Natural Science Foundation of China under Award Number 32100515 and 10.13039/501100004853CUHK direct grant for research under Award Numbers 2022.080 and 2025.031. KYY is supported by 10.13039/100000054National Cancer Institute of the 10.13039/100000002National Institutes of Health under Award Numbers P30CA030199 and R01CA287114, National Institute on Aging of the National Institutes of Health under Award Numbers R01AG085498 and U54AG079758, National Institute of General Medical Sciences of the National Institutes of Health under Award Number R21GM159319, and internal grants of Sanford Burnham Prebys Medical Discovery Institute. The content is solely the responsibility of the authors and does not necessarily represent the official views of the National Institutes of Health.

## CRediT authorship contribution statement

**Kevin Y. Yip:** Writing – review & editing, Writing – original draft, Project administration, Methodology, Investigation, Funding acquisition, Formal analysis, Conceptualization. **Qin Cao:** Writing – review & editing, Writing – original draft, Project administration, Methodology, Investigation, Funding acquisition, Formal analysis, Conceptualization. **Zhenghao Zhang:** Writing – review & editing, Methodology, Investigation, Formal analysis, Conceptualization. **Chen Miao:** Writing – review & editing, Methodology, Investigation, Formal analysis. **Daniel Rebibo:** Writing – review & editing, Investigation, Formal analysis. **Jiamin Chen:** Writing – review & editing, Methodology, Investigation, Formal analysis. **Sin-Hang Fung:** Writing – review & editing, Methodology, Formal analysis. **Haoran Wu:** Writing – review & editing, Formal analysis. **Stephen Kwok-Wing Tsui:** Writing – review & editing, Formal analysis. **Alfred Sze-Lok Cheng:** Writing – review & editing, Formal analysis. **Sanju Sinha:** Writing – review & editing, Formal analysis.

## Declaration of Competing Interest

The authors declare that they have no competing interests.

## Data Availability

The complete set of answers generated by all LLMs across all tasks, along with the manually curated results from our previous knowledgebase, are provided in Supplementary Materials. Examples of correct, partially correct, and incorrect answers produced by the LLMs are presented in Supplementary Table 4**-**Supplementary Table 6. The reproducible code and all the data used in this study are available at https://github.com/itachimigi/ici-llm and https://doi.org/10.5281/zenodo.15687725.
