# Supplementary material for: Developing foundations for biomedical knowledgebases from literature using large language models – A systematic assessment
Source: Comput Struct Biotechnol J. 2025 Jul 24;27:3299–306. doi: 10.1016/j.csbj.2025.07.042 (PMC12329539; doi:10.1016/j.csbj.2025.07.042)

**SUPPLEMENTARY MATERIALS**

**SUPPLEMENTARY TEXT**

**Scoring criteria for evaluation tasks**

Here we provide some examples to illustrate how our human inspectors determined the score of a contestant’s answer for each of the tasks.

1. Name of biomarker/prediction model

Instead of just the name of the predictor, we expected a concise yet informative full name that describes the associated biomarkers or meaning of the abbreviation or acronym. For instance, the answer “PSscore” in Paper 7 received 0.5, because the answer was partially lacking since we expected the answer to describe what “PSscore” stands for. In this case, “PSscore” refers to the Pyroptosis regulation patterns score.

Common/interesting issues: When the paper introduces variations of a predictor model, or compares the primary predictor with different predictors, LLMs may be limited to responding with one of the mentioned predictors (may not necessarily be the primary predictor), which may also lead to distortion of answers for all other questions.

1. Input data required and measurement methods

We expected the response to contain the correct input data required and measurement methods. However, if the response included data-level information, the correctness of such data should also be graded. For instance, in Paper 1, GPT-4o (zero-shot) produced the correct response (“PD1+ granulocyte percentage | Cytofluorimetric Test”; “|” separates the responses for “input data required” and “measurement methods”) and received a full score (score=1|1), while GPT-4o (few-shot) generated the correct response for “measurement methods” (“Flow Cytometry”, score=1) but not for “input data required” (“Protein Level”, score=0). The reason is that the input data required to compute the PD1+ granulocyte percentage should be at the cell level, as obtained from flow cytometry, rather than the protein level. Although the specific name of the measurement method is cell-level flow cytometry, responses that only referred to it as the general cytofluorimetric test were also considered acceptable.

Common/interesting issues: The responses may include extra data information mentioned in the paper, despite it not being directly required by the predictor.

1. Biosamples required

We expected the response to contain the exact and specific biosample types needed for the predictor computation. For instance, in Paper 2, because the analysis required obtaining germline normal DNA to analyze HLA-I homozygosity, the description should specify “normal tissue” rather than just saying “blood sample” or “saliva sample”. The answer “normal DNA” received 0.5, because it addressed the “normal” information, but DNA was not a biosample type.

1. Cancer types tested

We expected the response to contain all the specific cancer types for all the involved datasets, although organ level was not required. However, if the response included organ-level information, the correctness of such data should also be graded.

1. Species tested

We expected the response to contain all the specific species for all the involved datasets.

1. ICI types and specific drugs administered

We expected a thorough summary of the treatments containing all the involved ICI types and specific drugs administered. The response should detail all relevant specific ICI types, indicating their use as individual or combinatorial therapies, and clearly state any corresponding drug information, including whether the ICIs were combined with other non-ICI therapies. For example, the response “'anti-PD-1 | Nivolumab; anti-CTLA-4 | Ipilimumab” in Paper 4 received both 0.5 for the “ICI types” and “specific drugs administered” parts. This response was partially correct for both parts, as we expected a clear description showing the combination of the ICI types and drugs administered, like “anti-CTLA-4 + anti-PD-1 | Ipilimumab + Nivolumab”.

Common/interesting issues: Challenges were especially encountered in obtaining information on combinatorial therapies.

1. Treatment outcome variables

We expected the response to contain the specific efficacy endpoints that were assessed, such as objective response rate, progression-free survival, or overall survival.

Common/interesting issues: Some responses may report response rates only, while survival analyses were omitted.

1. Association direction with ICI efficacy

We expected the response to include a clear assessment of the association between the predictors of interest and the efficacy outcomes, including whether the association was positive, negative, or dependent on other factors. Any positive or negative correlations should be explicitly stated, with the direction of the effect matching the provided predictor name.

Common/interesting issues: Difficulties may arise when the association was more complex or contingent on other variables.

1. Features derived from input data

We expected that the LLMs could extract the features accurately and precisely. Additionally, the methods used for feature processing and selection, including approaches like grouping by specific features and identifying the most important ones, should be clearly detailed. For instance, the answer “Expression levels of 33 pyroptosis-related genes” in Paper 7 received 0.5/3=0.17. The correct answer should explain only the top two principal components (PCs) of the overlapping genes with the 33 pyroptosis genes that were used to calculate the PSscore. Thus, the feature alone received a score of 0.5 out of 1, while the feature processing/selection approach received a score of 0 out of 2.

1. Description of biomarker/prediction model

We expected that the response contained a comprehensive overview of both the correct model type and the key results associating with ICI efficacy.

Common/interesting issues: The description of the model type was often vague in the response; one possible difficulty was that the model type defined in the prompt was based on our knowledge. In LLMs’ responses, additional information was frequently provided about the predictive model, including evaluations with supplementary settings or target outcomes that may not directly relate to the primary results of the predictor or ICI therapy.

1. Details of tested cohort

If the cohort was not constructed by the current study being analyzed, but based on data from previous studies, we expected the response to include accurate data source information, which was worth 0.5 points. Additionally, the total number of patients included in the analysis should be clearly stated, which was also worth 0.5 points.

**Related efforts in LLM-based literature curation**

Beyond our assessment of general-purpose LLMs, the field is rapidly advancing through diverse, specialized approaches; pioneering efforts in agent-based systems, multi-LLM frameworks, and domain-specific models. For instance, agent-based systems like Crow from FutureHouse use structured workflows for complex literature searches, while multi-LLM frameworks such as ArticleLLM combine the capabilities of multiple models to improve insight extraction.

The domain-specific direction is equally promising, with highly focused tools ranging from RNAcentral LitSumm, which produces automated summaries for non-coding RNA genes, to advanced clinical models like Med-PaLM 2, showing the value of tailoring models to specific tasks. Despite their high performance, the broader application of some specialized systems faces practical limitations. For example, technical specifications can be a constraint; certain medical-specific models like Meditron lack the large context window (>16k tokens) required to process full-text scientific articles. Furthermore, the restricted access to leading proprietary models hinders their widespread adoption and independent validation.

Therefore, our findings establish a crucial baseline for general-purpose models while highlighting the field’s promising trajectory. The future of reliable, automated literature curation will likely not depend on a single solution but on a synthesis of these approaches.

**SUPPLEMENTARY TABLES**

**Supplementary Table 1 Examples that demonstrate inconsistency between scores produced by manual checking and common evaluation measures.**


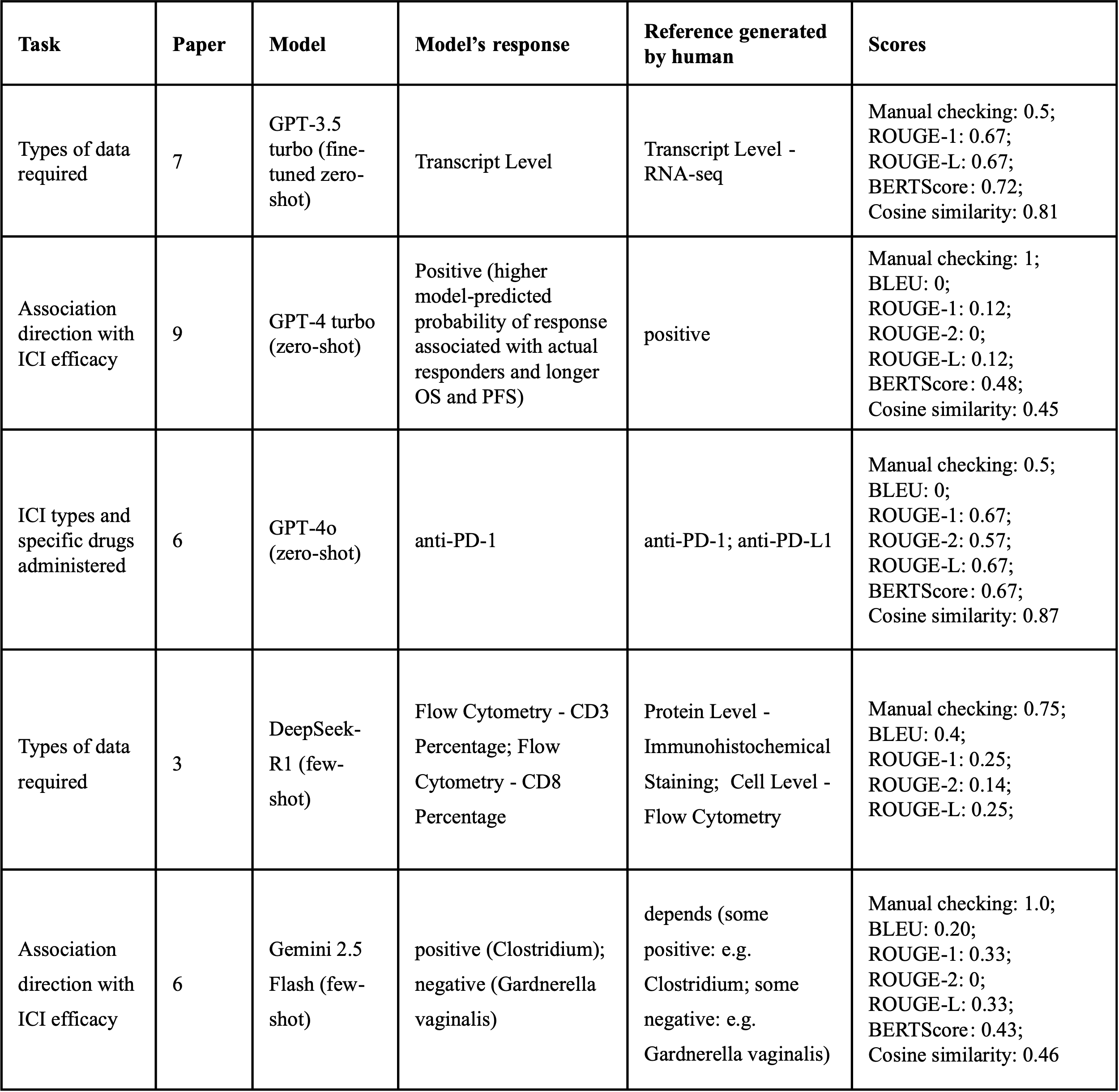


**Supplementary Table 2 Information about the 29 publications involved in this study.**


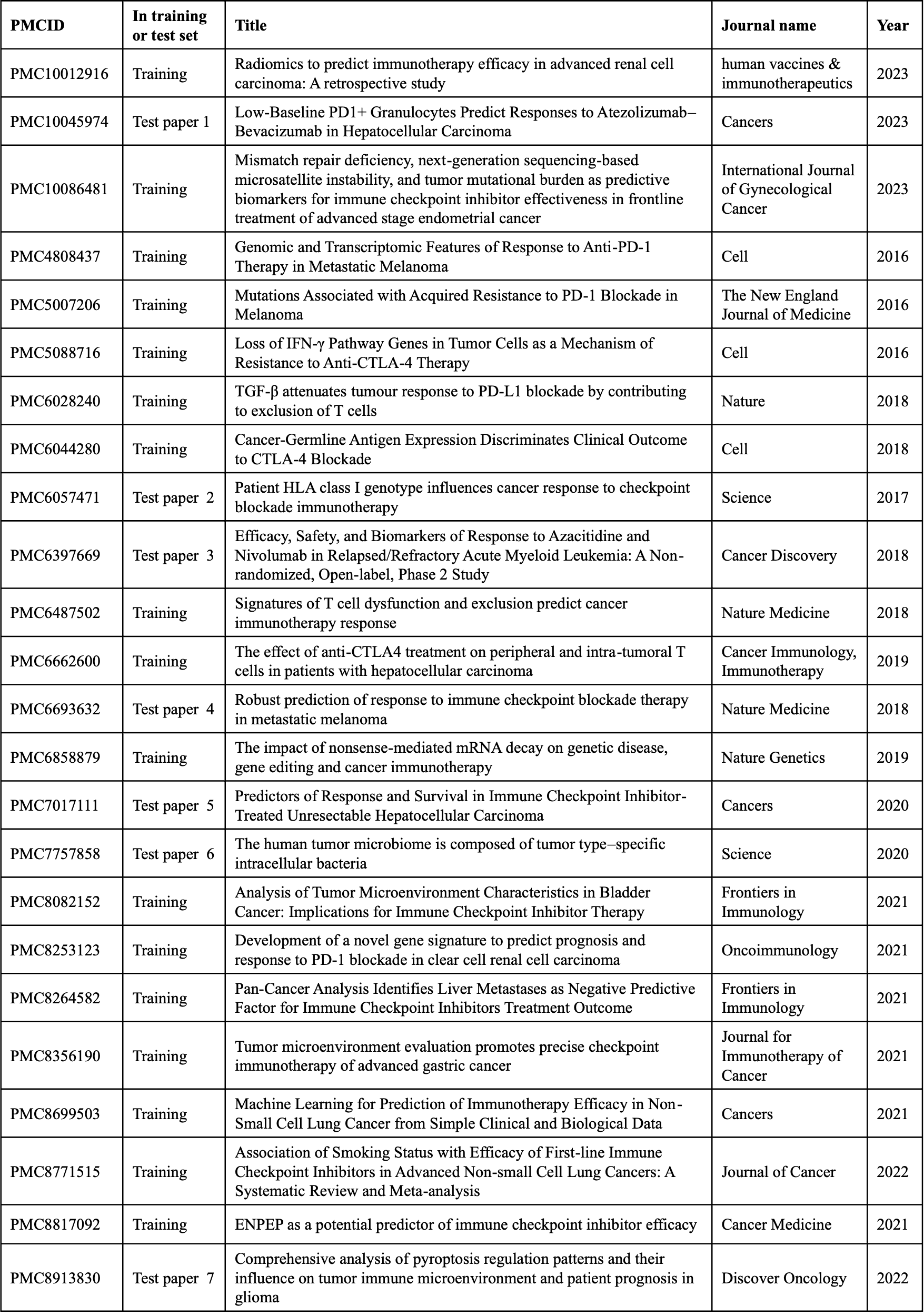


*
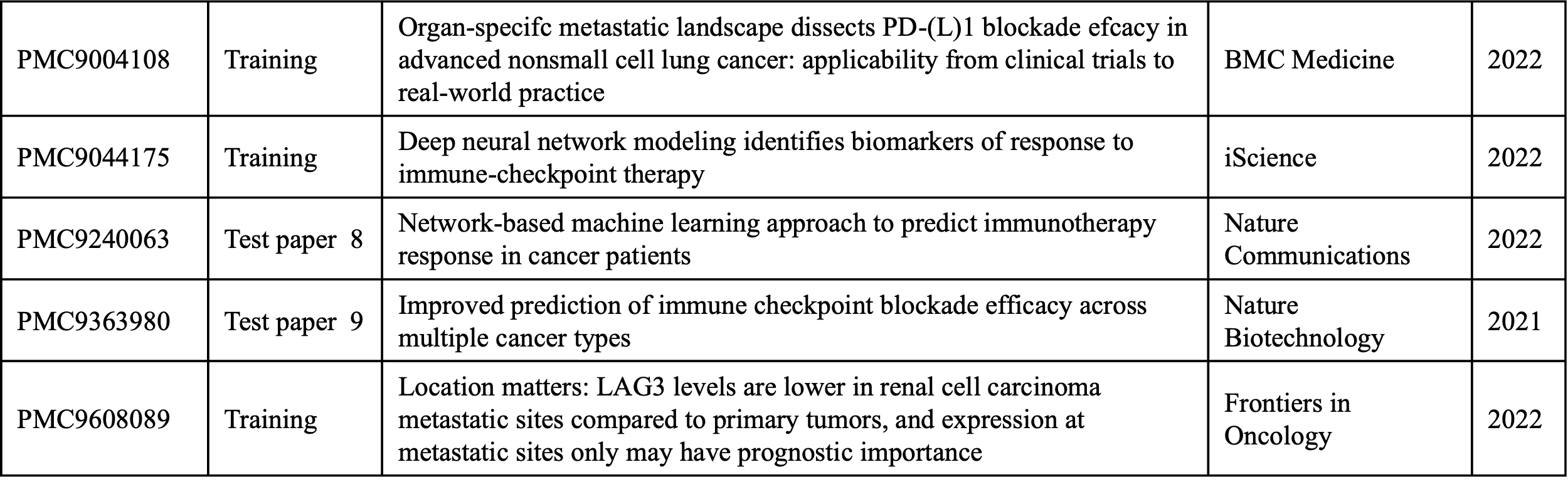
*

**Supplementary Table 3 Information about the tested LLMs, including context window, training data cutoff date, and pricing for different settings.**


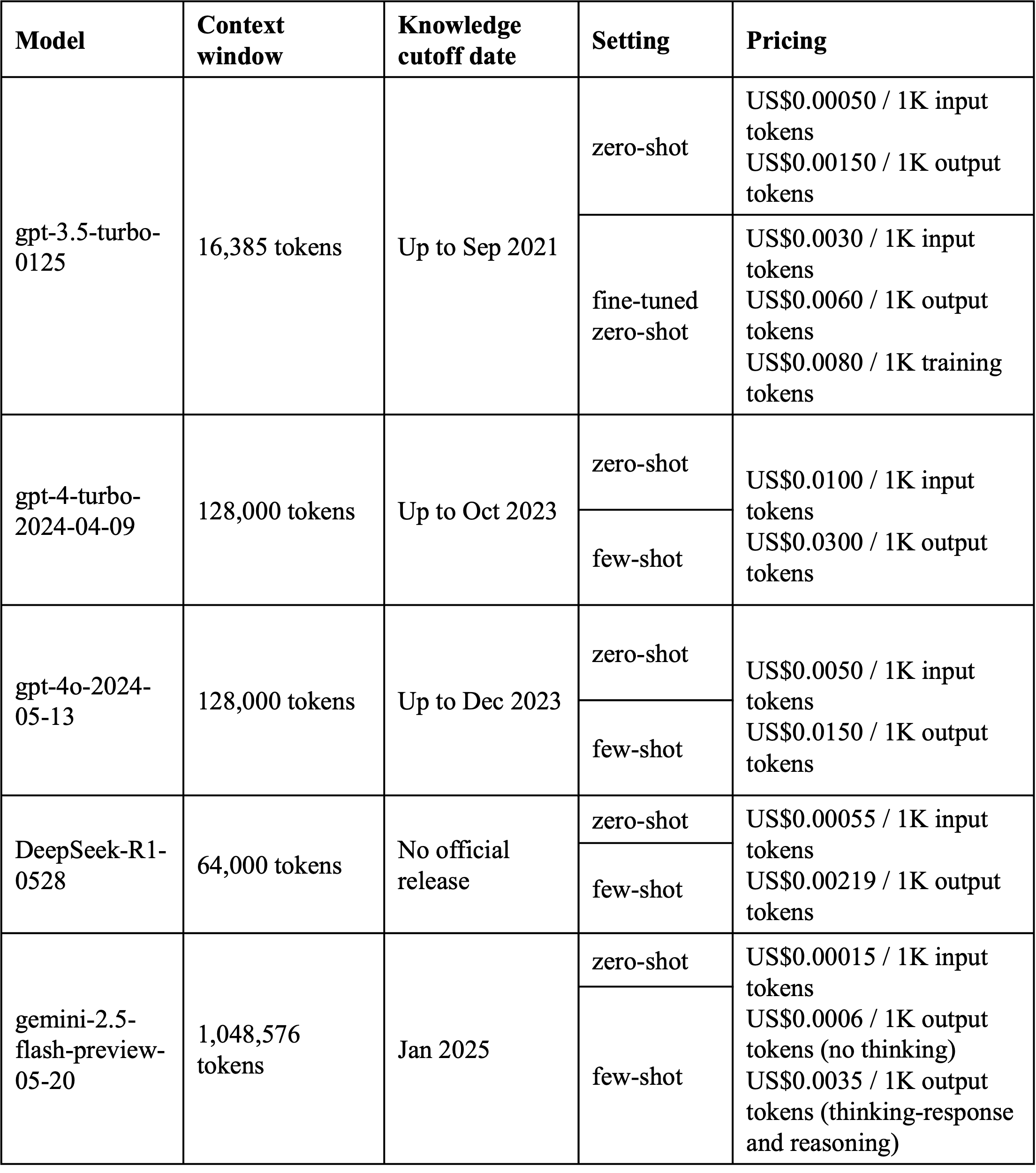


**Supplementary Table 4 Examples of correct answers generated by the LLMs.**


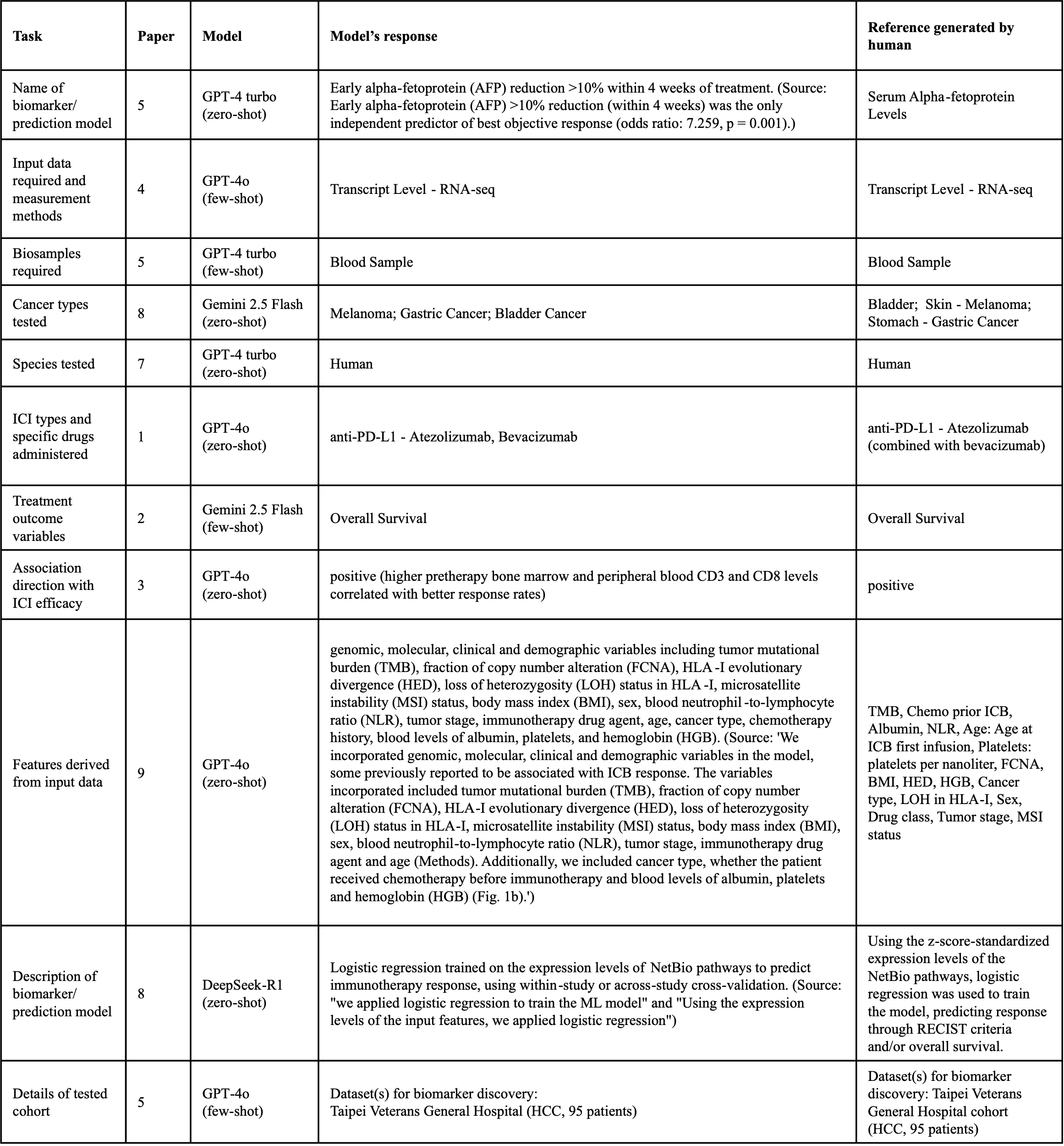


**Supplementary Table 5 Examples of partially correct answers generated by the LLMs.**


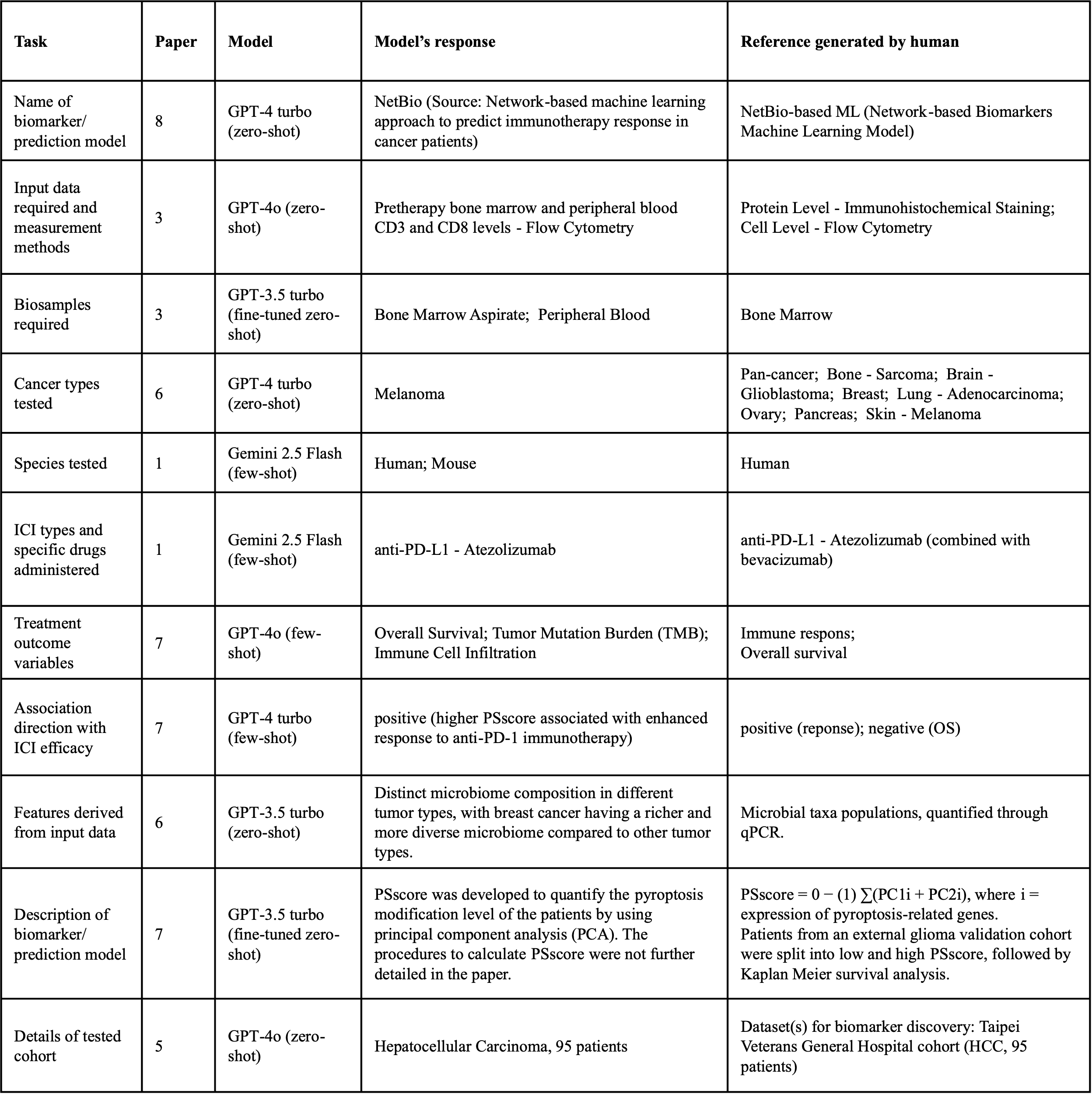


**Supplementary Table 6 Examples of incorrect answers generated by the LLMs.**

**
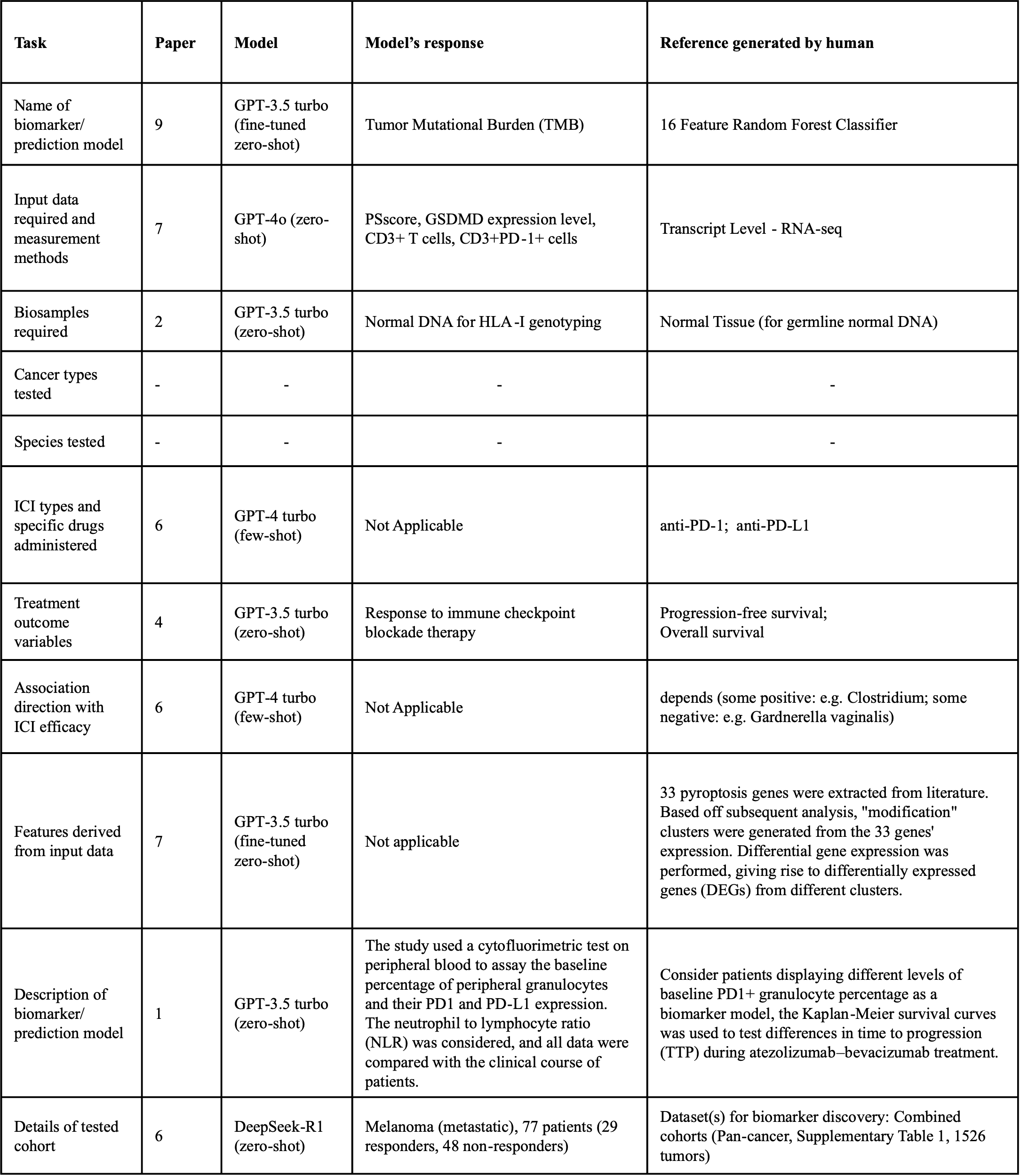
**

**SUPPLEMENTARY FIGURES**

**
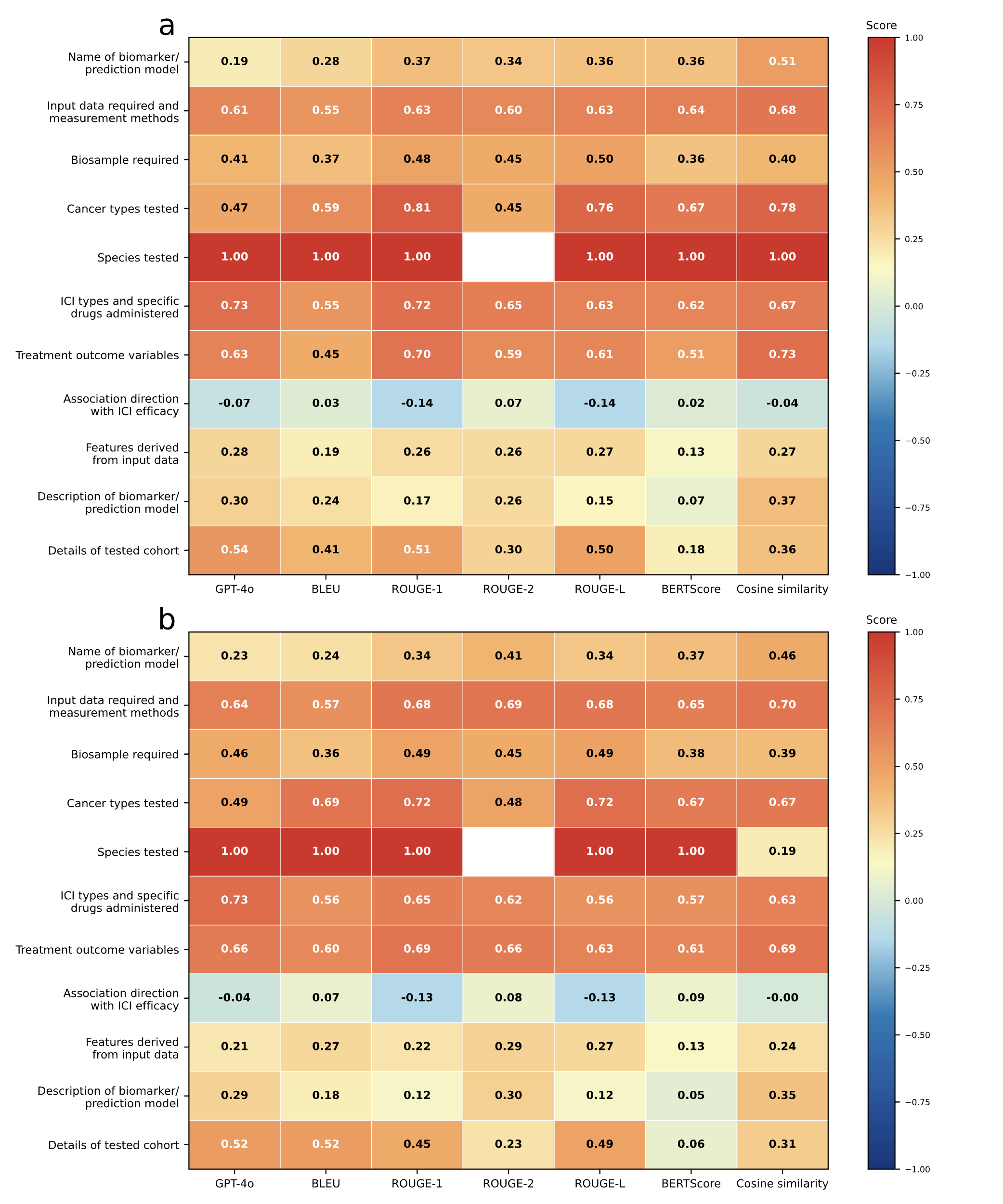
**

**Supplementary Figure 1.** **Correlations between the manual checking derived scores and the scores produced by the two other evaluation strategies.** Both the numbers and colors represent the Pearson correlation coefficients (**a**) and Spearman correlation coefficients (**b**) between the manual checking scores and the scores produced by a specific evaluation measure (the columns) on a specific task (the rows). “GPT-4o” denotes the Evaluation Strategy 3, namely asking an LLM (i.e., GPT-4o) to judge the similarity between an answer and the reference.


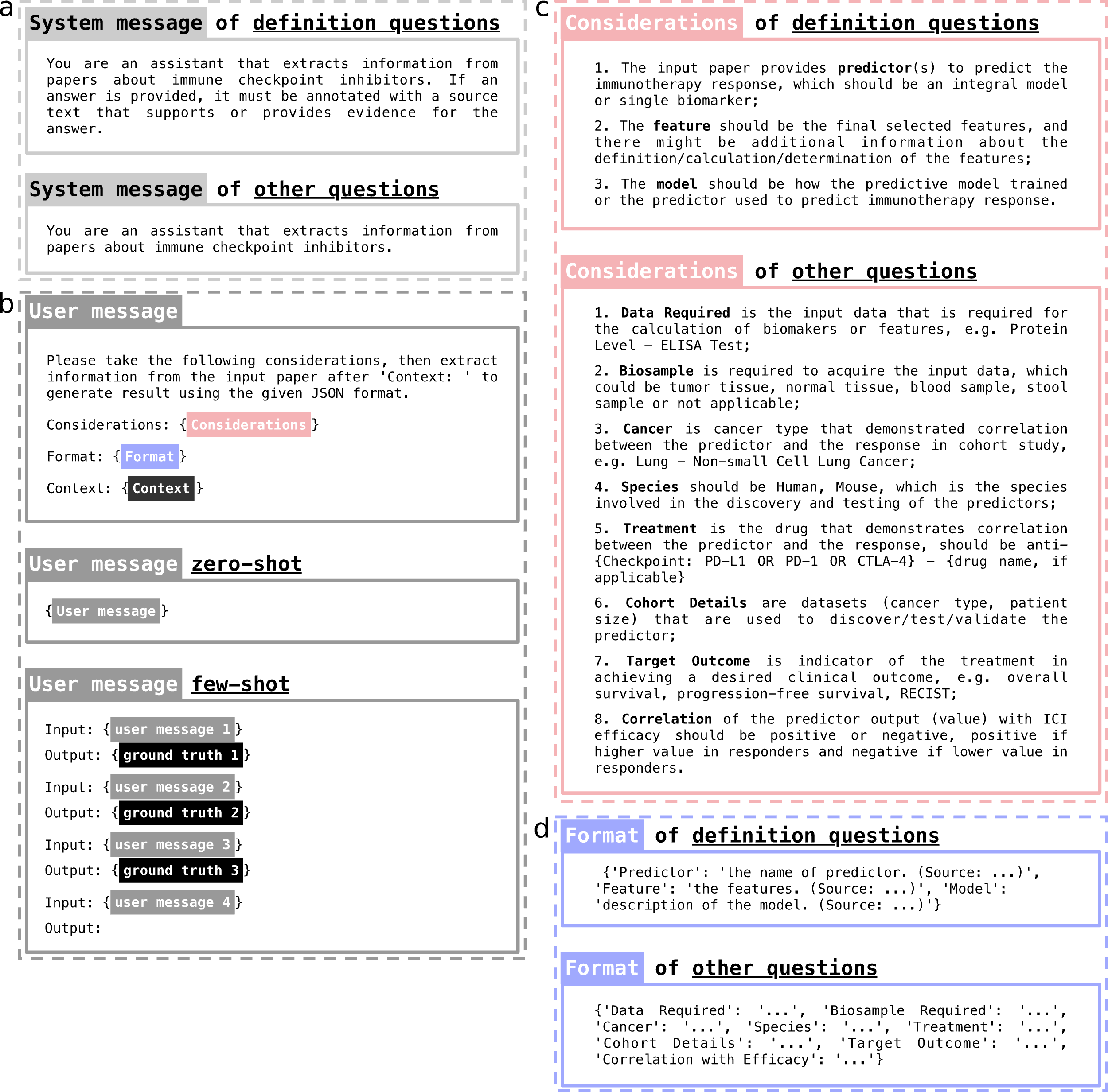


**Supplementary Figure 2. Our prompt designs.** Each prompt consists of different components, including the system message (**a**) and the user message (**b**), the latter of which contains the consideration (**c**), the format (**d**) and the context. The design of each component depends on the type of tasks (definition tasks versus other tasks) and the prompting mode (zero-shot versus few-shot). The prompts in this figure are the verbatim prompts used in this study.


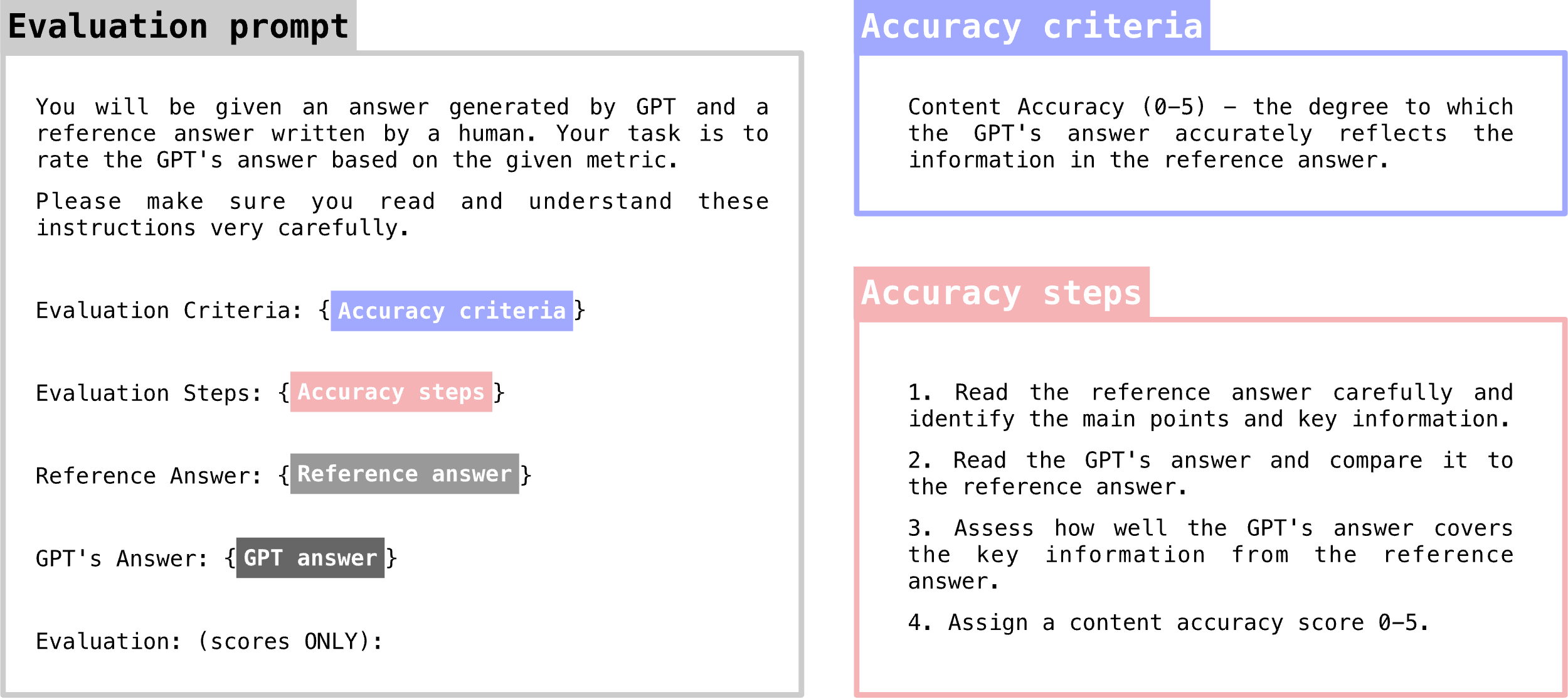


**Supplementary Figure 3.** **The prompt designed for asking GPT-4o to evaluate the answer of a contestant against the manually curated reference.**


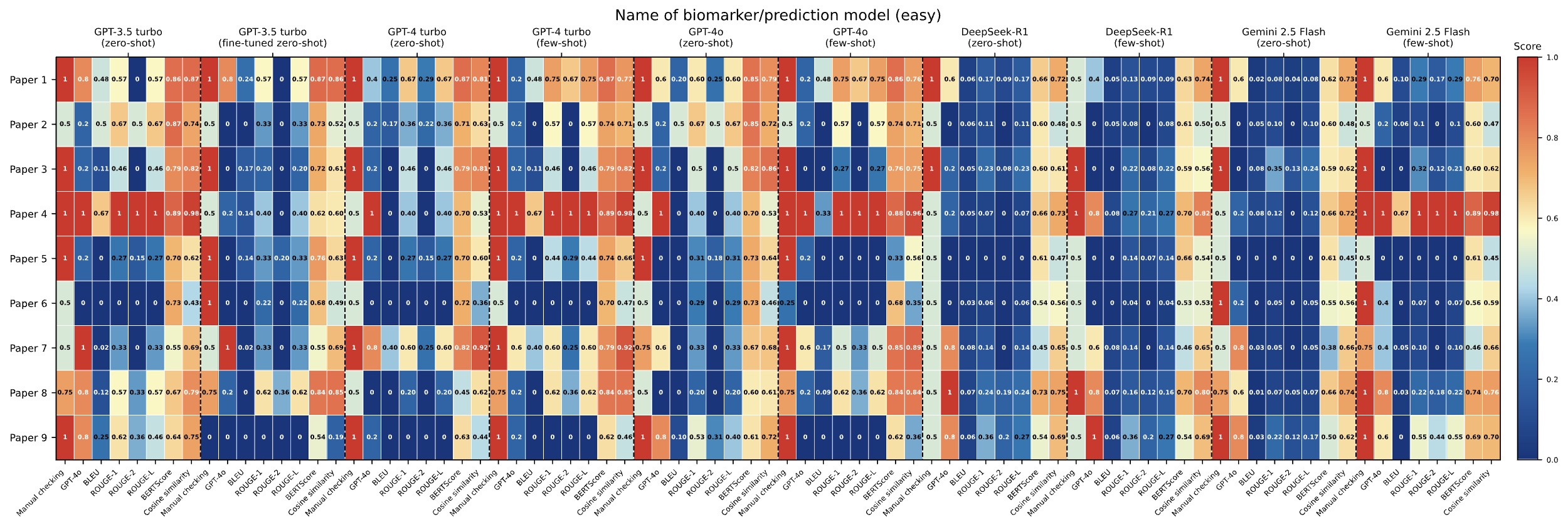


**Supplementary Figure 4.** **The complete set of scores produced by all evaluation strategies across all tasks.** The figure shows the following items: Heatmaps – representing tasks; columns of heatmaps – corresponding to evaluation measures; column groups – corresponding to contestants; rows – corresponding to papers. For the “Species tested” task, all the reference answers are single words (i.e., “Human”). In such instances, where there are no bigrams to compare, the ROUGE-2 scores are calculated as zeros. (Please also see additional 5 pages of the figure.)


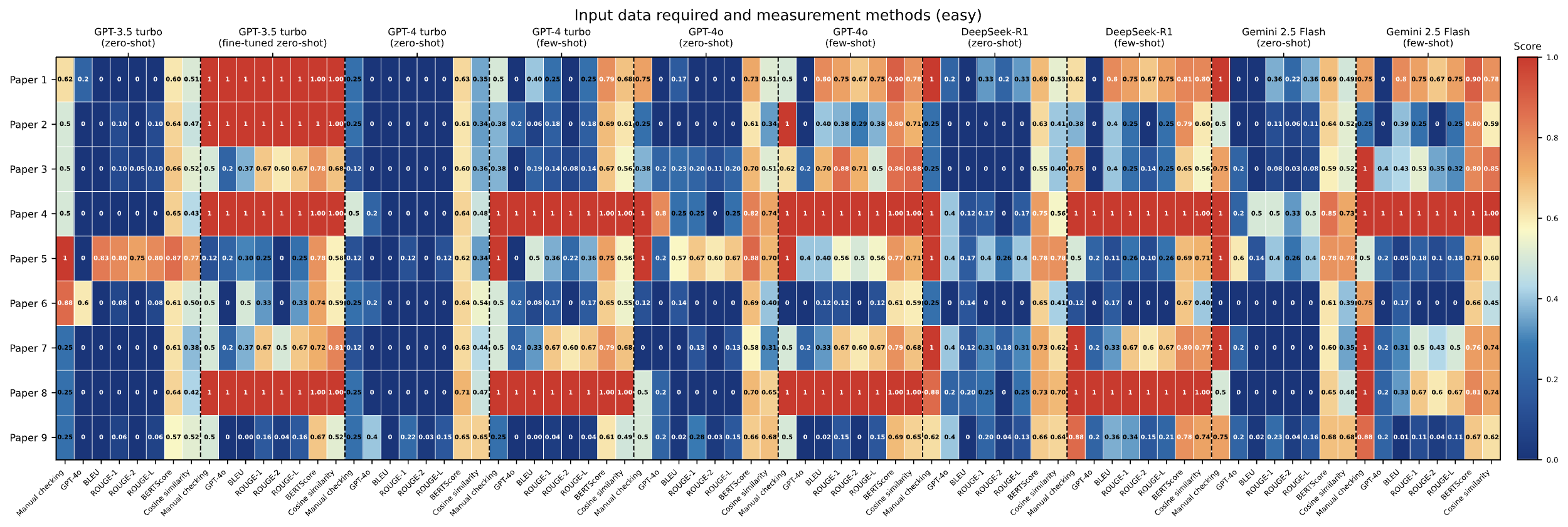

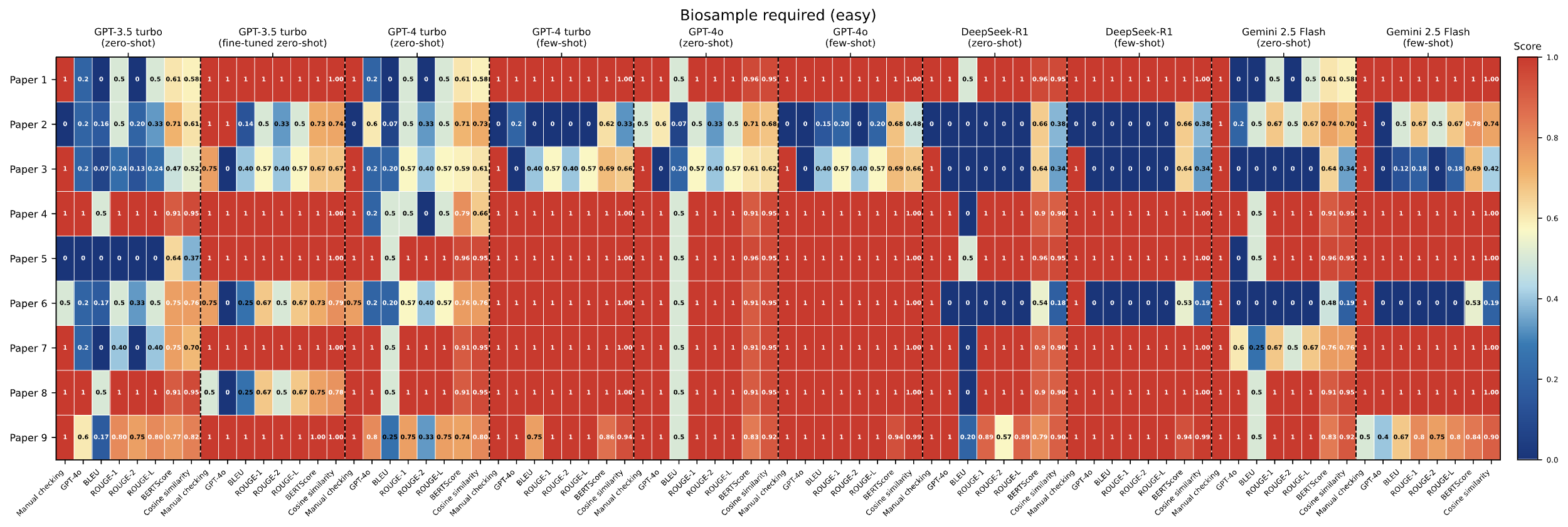

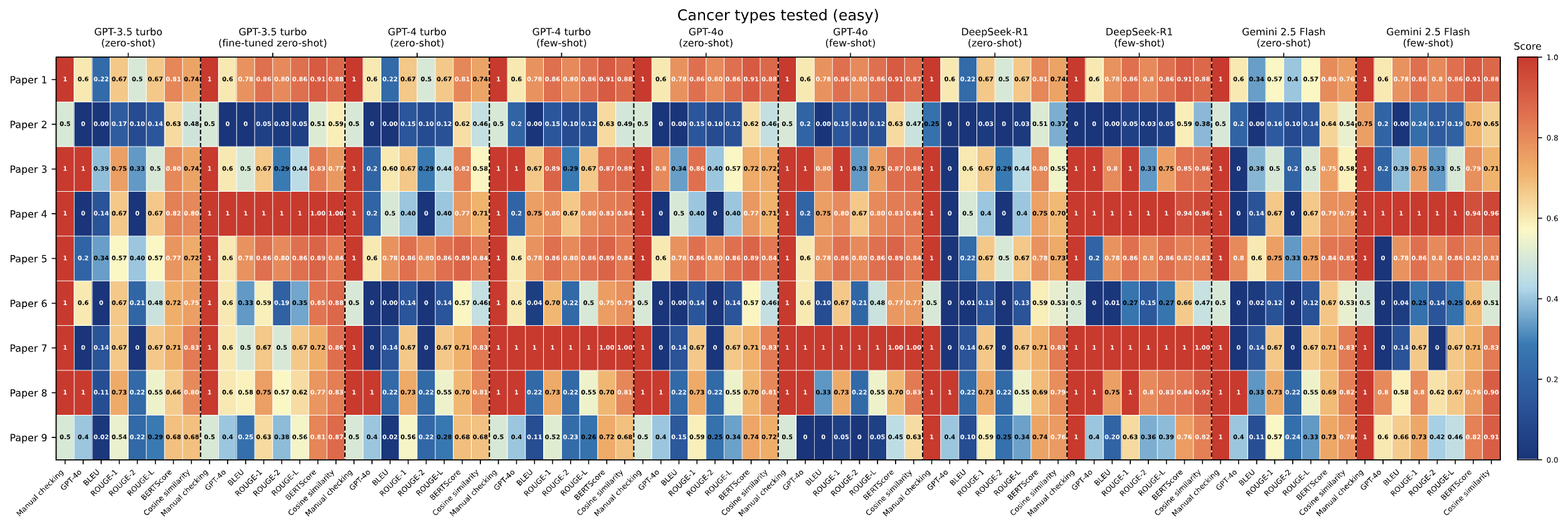

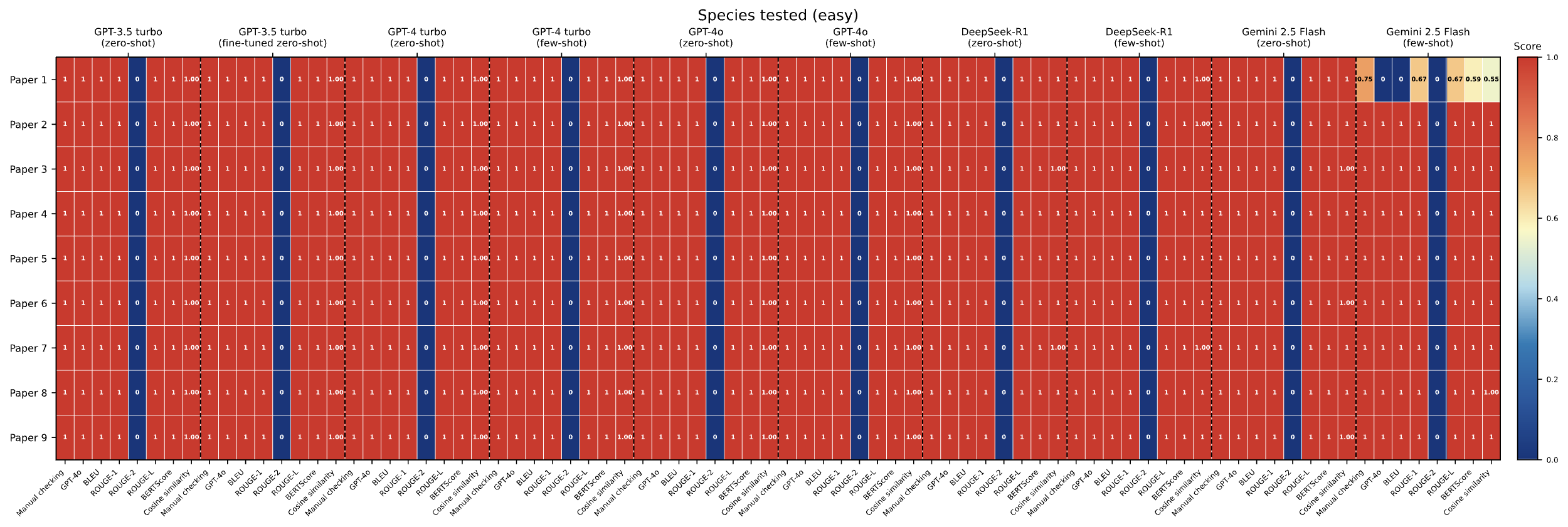


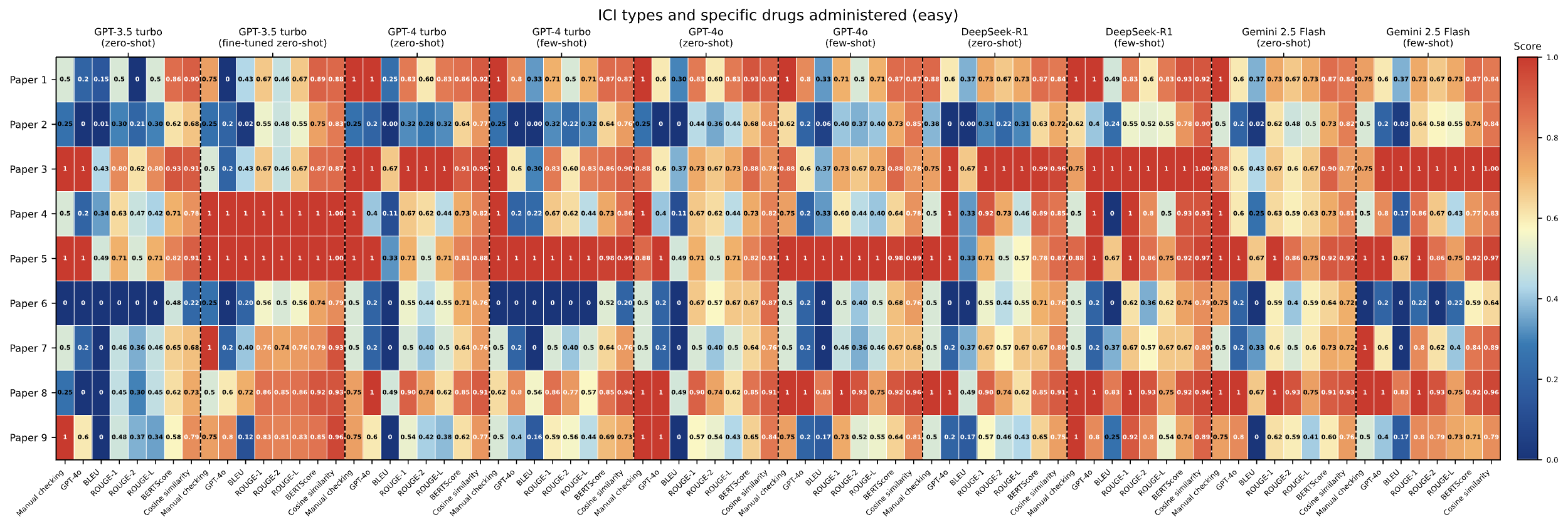

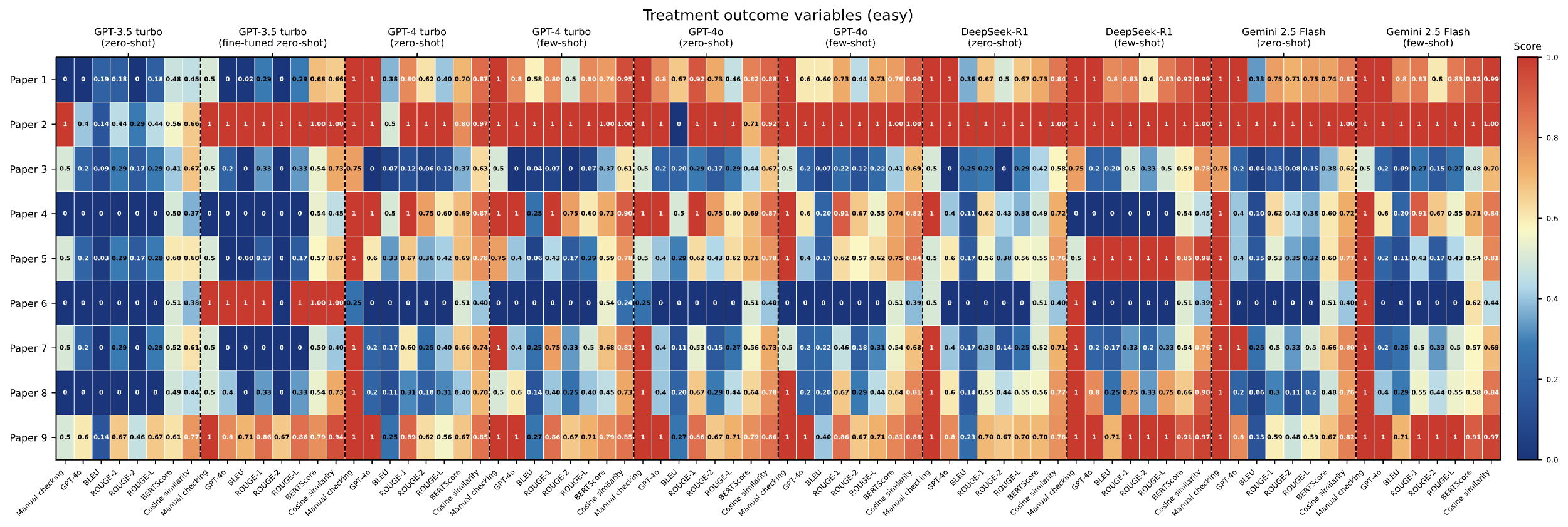

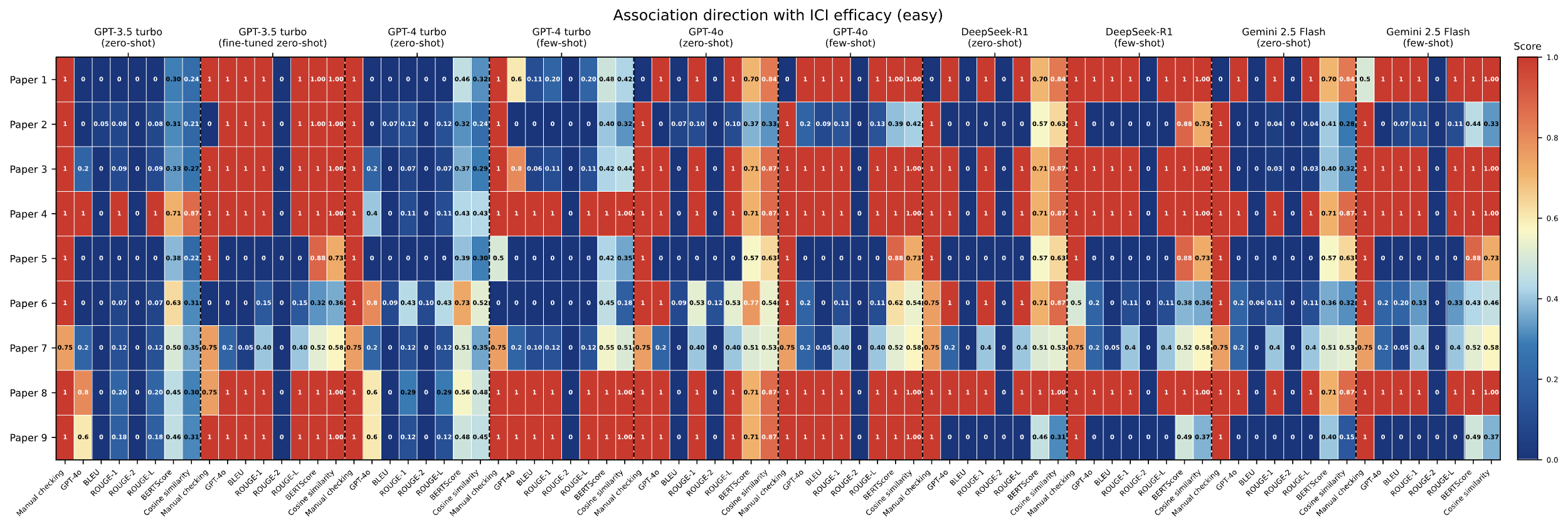

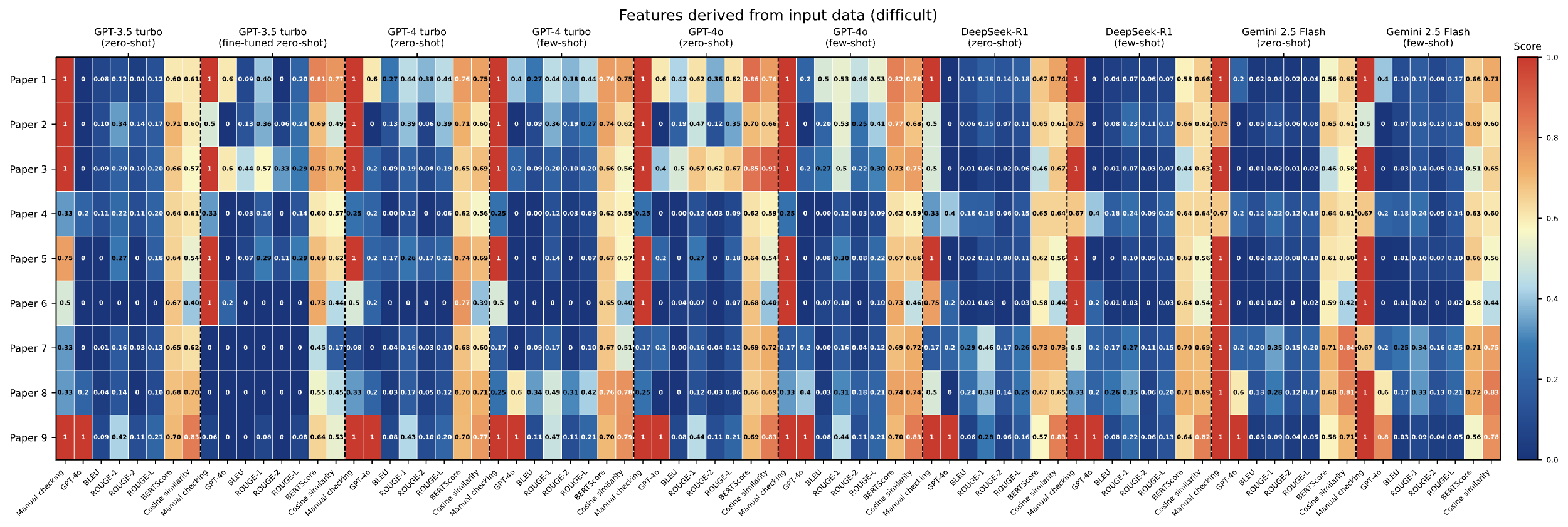


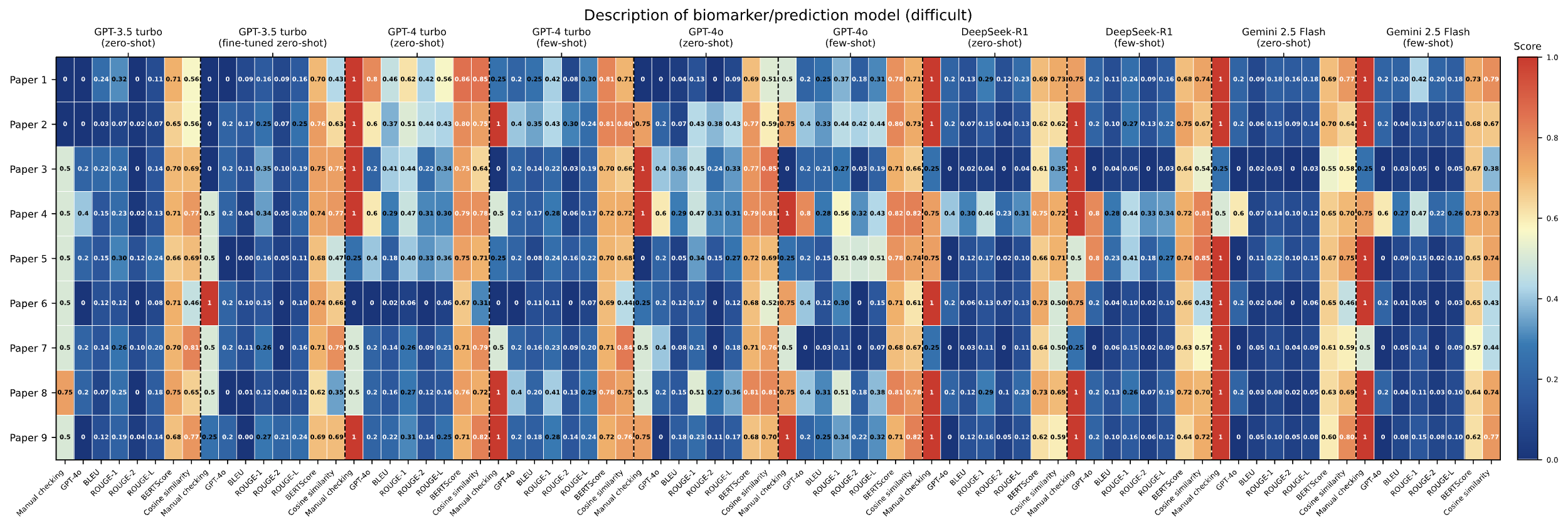

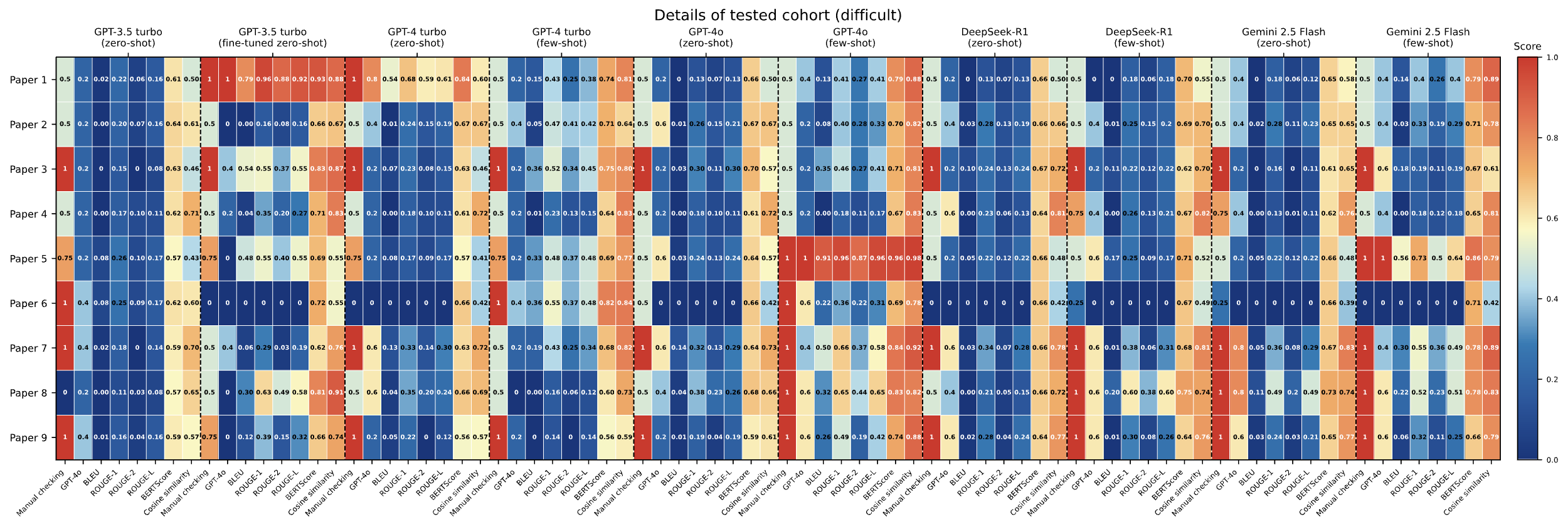

Supplement: Supplementary file 1 — Supplementary material [file mmc1.docx]
